# Influence of an immunodominant herpes simplex virus type 1 CD8^+^ T cell epitope on the target hierarchy and function of subdominant CD8^+^ T cells

**DOI:** 10.1371/journal.ppat.1006732

**Published:** 2017-12-04

**Authors:** Benjamin R. Treat, Sarah M. Bidula, Srividya Ramachandran, Anthony J. St Leger, Robert L. Hendricks, Paul R. Kinchington

**Affiliations:** 1 Molecular Virology and Microbiology Graduate Program, University of Pittsburgh, Pittsburgh, Pennsylvania, United States of America; 2 Department of Ophthalmology, University of Pittsburgh, Pittsburgh, Pennsylvania, United States of America; 3 Immunology Graduate Program, University of Pittsburgh, Pittsburgh, Pennsylvania, United States of America; 4 Department of Immunology, University of Pittsburgh, Pittsburgh, Pennsylvania, United States of America; 5 Department of Microbiology and Molecular Genetics, Pittsburgh, Pennsylvania, United States of America; University of Washington, UNITED STATES

## Abstract

Herpes simplex virus type 1 (HSV-1) latency in sensory ganglia such as trigeminal ganglia (TG) is associated with a persistent immune infiltrate that includes effector memory CD8^+^ T cells that can influence HSV-1 reactivation. In C57BL/6 mice, HSV-1 induces a highly skewed CD8^+^ T cell repertoire, in which half of CD8^+^ T cells (gB-CD8s) recognize a single epitope on glycoprotein B (gB_498-505_), while the remainder (non-gB-CD8s) recognize, in varying proportions, 19 subdominant epitopes on 12 viral proteins. The gB-CD8s remain functional in TG throughout latency, while non-gB-CD8s exhibit varying degrees of functional compromise. To understand how dominance hierarchies relate to CD8^+^ T cell function during latency, we characterized the TG-associated CD8^+^ T cells following corneal infection with a recombinant HSV-1 lacking the immunodominant gB_498-505_ epitope (S1L). S1L induced a numerically equivalent CD8^+^ T cell infiltrate in the TG that was HSV-specific, but lacked specificity for gB_498-505_. Instead, there was a general increase of non-gB-CD8s with specific subdominant epitopes arising to codominance. In a latent S1L infection, non-gB-CD8s in the TG showed a hierarchy targeting different epitopes at latency compared to at acute times, and these cells retained an increased functionality at latency. In a latent S1L infection, these non-gB-CD8s also display an equivalent ability to block HSV reactivation in *ex vivo* ganglionic cultures compared to TG infected with wild type HSV-1. These data indicate that loss of the immunodominant gB_498-505_ epitope alters the dominance hierarchy and reduces functional compromise of CD8^+^ T cells specific for subdominant HSV-1 epitopes during viral latency.

## Author summary

Most HSV-1 disease, including potentially blinding herpes stromal keratitis, results from sporadic reactivation of latent HSV-1 within sensory ganglia. Latently infected ganglia of humans and mice are associated with a persistent immune infiltrate of CD4^+^ and CD8^+^ T cells, with ganglionic CD8^+^ T cells capable of blocking HSV-1 reactivation from *ex vivo* cultures of latently infected ganglia. Here we show that in the absence of CD8^+^ T cells that recognize a single highly immunodominant epitope, the CD8^+^ T cells specific for the remaining 19 subdominant viral epitopes are not only numerically enhanced, but show more function within latently infected ganglia. We propose this work could lead to strategies that broaden and expand the functional CD8^+^ T cell repertoire within latently infected sensory ganglia, which may reduce the incidence of HSV-1 reactivation and recurrent disease.

## Introduction

Primary herpes simplex virus type 1 (HSV-1) infection at peripheral mucosal sites leads to infection of innervating axonal termini, retrograde virus transport to nuclei of sensory and sympathetic neurons, and the establishment of a persistent latent state that is then maintained for the life of the host[1–3]. During latency, numerous factors, such as viral and host encoded miRNAs [4–6]and host epigenetic regulation [7–9], contribute to a repression of most lytic viral genes. During latency, abundant transcription is limited to a family of non-coding RNAs, the latency-associated RNA transcripts (LATs), which have been proposed to have multiple activities that promote latency and survival of the infected neurons [10, 11]. Sporadic or induced full HSV reactivation in humans can result in virus delivery to the periphery and development of recurrent disease. Recurrence in the eye is particularly problematic, since it may initiate a recurring immune-mediated herpes stromal keratitis (HSK) that causes progressive corneal scarring and opacity. Indeed, HSK is the most frequent infectious cause of blindness in the developed world[12].

Many lines of evidence now strongly suggest that lytic gene expression is not fully repressed during latency, but is rather in a dynamic state where sporadic lytic viral RNA and protein expression can occur in the neuron without virus production. It has been proposed that such sporadic HSV gene expression is largely outside of the typical α, β, γ cascade seen in productive infections [4, 8, 13–16]. A key decision is whether such sporadic events revert to a repressive state or subsequently progress to virus production. Evidence suggests that such chronic and sporadic viral gene expression in the latently infected ganglia is immune recognized, particularly by a persistent resident ganglionic CD8^+^ T cell population [17–19]. Indeed, the mouse model of HSV-1 latency has been under particular scrutiny, with the initial viral occupancy of the ganglia accompanied by a large infiltration of immune cells, including both CD4^+^ and CD8^+^ T cells. This immune infiltrate peaks near the onset of latency and then rapidly contracts, leaving a persistent low-level infiltrate that is maintained for the life of the host. Persisting ganglionic immune infiltrates associated with HSV-1 latency have also been seen in other model species and in humans [20–24]. The ganglionic CD8^+^ T cells in mice show markers of an activated effector memory phenotype, which is capable of reducing HSV-1 reactivation events in *ex vivo* cultures of latently infected ganglia[19, 25]. These observations promote a suspected role of adaptive cellular immunity in regulating the HSV-1 latent/lytic decisions *in vivo*. As such, gaining insights that allow improvement of the size, antigenic diversity, or the functional state of the ganglionic immune infiltrate may help increase protection from HSV-1 reactivation and subsequent disease [26].

The C57BL/6 (B6) mouse ocular HSV infection model has been particularly useful to explore cellular CD8^+^ T cell directed immunity, because the entire HSV-1 specific CD8^+^ T cell target repertoire has been described [27]. CD8^+^ T cells recognize short peptides of processed proteins (epitopes) that are presented bound to major histocompatibility complex class I (MHC-I) at the cell surface. In B6 mice, the CD8^+^ T cell repertoire developed to HSV-1 is highly skewed; a single immunodominant epitope on the essential viral glycoprotein B (gB) accounts for approximately half of all HSV-1 specific CD8^+^ T cells (gB-CD8s). These gB-CD8s are directed to amino acids 498–505 of gB (SSIEFARL, henceforth referred to as gB_498-505_). The other HSV-1 specific CD8^+^ T cell populations (collectively termed non-gB-CD8s) recognize 19 additional subdominant viral epitopes on only 12 viral proteins [27]. The approximate 1:1 ratio of HSV gB-CD8s to non-gB-CD8s is maintained systemically, in the corresponding lymph nodes, the spleen, and in the TG during acute peak infiltrate and in the contracted population during latency [28, 29]. However, while gB-CD8s during HSV-1 latency show an activated and highly functional phenotype that responds to antigen stimulation, non-gB-CD8 populations show a partial loss of function, such that a significant fraction do not respond to antigen stimulation. Loss of function in the non-gB-CD8 population resembles functional exhaustion in that it is associated with increased expression of the inhibitory receptor programed death-1 (PD-1) and is regulated by IL-10 [30,31]. Thus, the TCR repertoire of functional CD8^+^ T cells narrows by a process akin to functional exhaustion of subdominant CD8^+^ T cells within the latently infected TG.

The CD8^+^ T cell dominance hierarchy seen in the C57Bl/6 model and the strong dominance of the HSV-1 gB_498-505_ epitope is remarkable. It is not clear why the gB_498-505_ epitope is so immunodominant; the position of an epitope within the dominance hierarchy can be influenced by many factors [32]. Theoretical host disadvantages to strong immunodominance include a less diverse TCR repertoire, and the potential for viral escape from CD8^+^ T cell control through mutations in the immunodominant epitope [32]. The combination of a strongly immunodominant gB_498-505_ epitope and a completely defined TCR repertoire now makes HSV-1 an excellent model to investigate the effect of removing an immunodominant epitope on the resulting CD8^+^ T cell repertoire and changes associated with the latent state. Here, we fully characterize this response in acute and latent HSV-1 infections.

## Results

### Mutations in HSV-1 gB_498-505_ prevent antigen recognition by gB-CD8s

**Fig 1:**
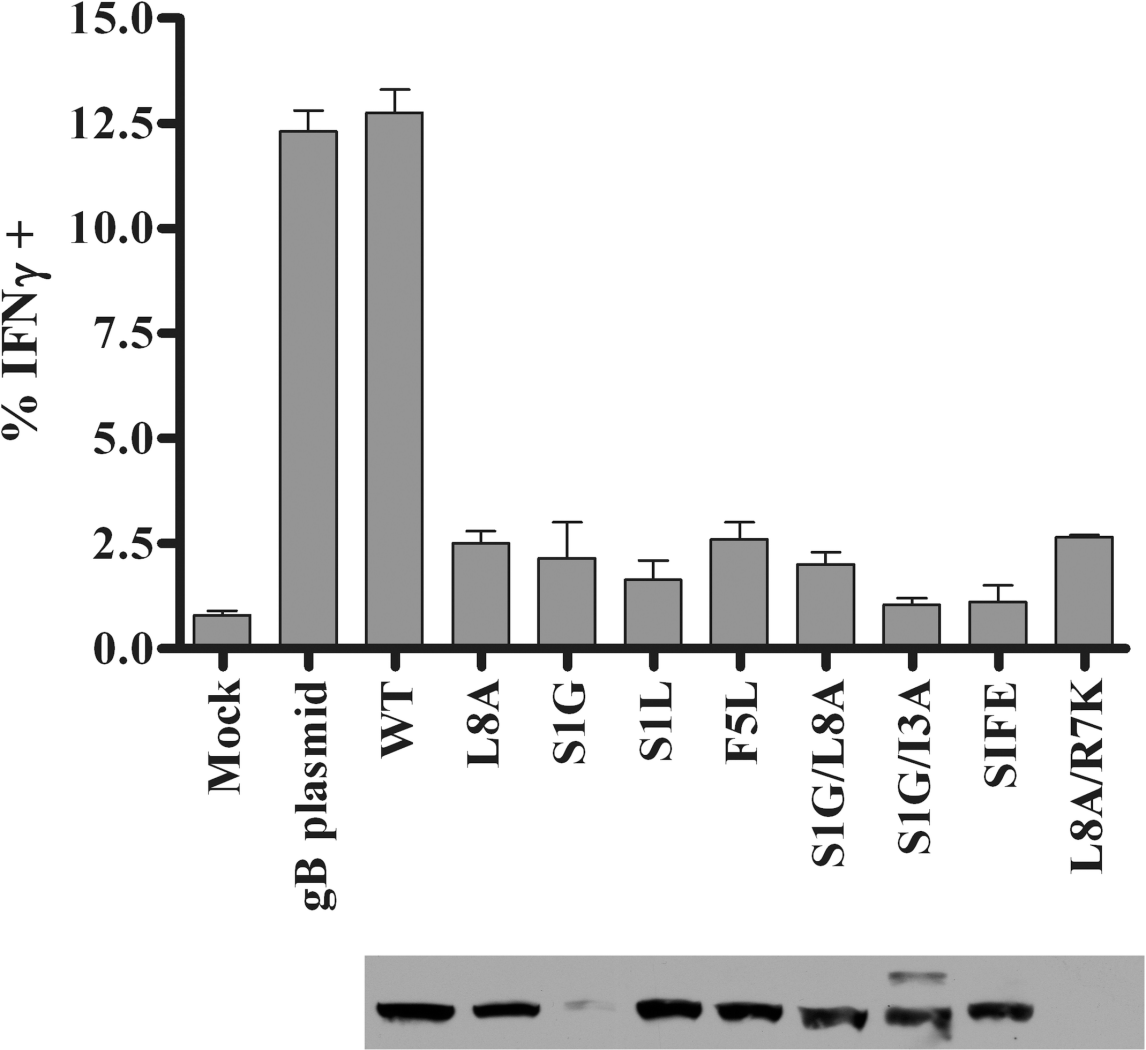
Mutant gB proteins and recognition by gB-CD8s. Untreated B6WT3 cells (mock) or cells transfected with plasmids to express WT gB or gB_498-505_ epitope mutants were incubated for eighteen hours. The labeling of the mutations is as depicted in Table 1; WT is a plasmid which went through the mutagenesis procedure without changes. Cells were harvested for expression analysis by immunoblotting using a monoclonal gB-specific antibody (lower panel) or, in parallel, transfected cells were combined with 5x10^4^ gB-CD8s from an endogenously expanded clone and stimulated for 5 h in the presence of Brefeldin A. gB-CD8s were surface stained for CD45 and CD8, permeabilized, and stained for intracellular IFNγ. The graph depicts one of two representative experiments, with the mean percent of IFNγ^+^ cells (*n* = 2/group) and standard error of the mean (SEM) for each stimulation.

A series of gB mutants in the 498–505 amino acid region was generated and evaluated for recognition by gB-CD8s. The eight gB mutations (Table 1) included: mutations of the predicted MHC-I anchoring residues in the peptide (L8A, S1L, S1G, L8A/S1G, and L8A/R7K); mutations in the predicted T cell receptor binding region (F5L and S1G/I3A): and a mutation that changed the HSV-1 gB_498-505_ region to that of VZV, S1G/l3N/F5L/E4S (SIFE). The L8A mutation was previously reported (also referred to as L505A) in HSV-1 to abrogate gB-CD8 development upon flank skin infection of B6 mice [33]. We found that most of the eight mutant gB genes expressed a protein from plasmids that were detected by gB-specific antibodies of the size expected for gB (Fig 1). When each mutant gB protein was expressed in transfected B6WT3 fibroblasts, only wild-type (WT) gB could stimulate the production of intracellular IFNγ in an expanded population of gB-CD8s (Fig 1). While only a modest fraction of gB-CD8s showed activation, it was clear that all 8 mutations of the gB_498-505_ region abrogated gB-CD8 recognition.

**Table 1:** Primer sequences (complementary to the coding sequence) used in PCR to generate mutations in the gB epitope (SSIEFARL) region.

*Name*	*Resulting Mutation*	Reverse primers used*
**WT**	SSIEFARL (none)	5’ GTT G**TA CGT A**AA CTG CAG CCT GGC GAA CTC GAT GGA GGA GGT GGT CTT GAT GCG CTC CA 3’
**L8A**	SSIEFARA	5 ‘ GTT G**TA CGT A**AA CTG agc CCT GGC GAA CTC GAT GGA GGA GGT GGT CTT GAT GCG CTC CA 3’
**F5L**	SSIELARL	5’ GTT G**TA CGT A**AA CTG CAG CCT GGC cAA CTC GAT GGA GGT GGT CTT GAT GCG CTC CA 3’
**S1G**	GSIEFARL	5’ GTT G**TA CGT A**AA CTG CAG CCT GGC GAA CTC GAT GGA ccc GGT CTT GAT GCG CTC CA 3’
**S1L**	LSIEFARL	5’ GTT G**TA CGT A**AA CTG CAG CCT GGC GAA CTC GAT GGA caa GGT GGT CTT GAT GCG CTC CA 3’
**S1G/L8A**	GSIEFARA	5’ GTT G**TA CGT A**AA CGT agc CCT GGC GAA CTC GAT GGA ccc GGT GGT CTT GAT GCG CTC CA 3’
**S1G/I3A**	GSAEFARL	5’ GTT G**TA CGT A**AA CTG CAG CCT GGC GAA CTC Ggc GGA ccc GGT GGT CTT GAT GCG CTC CA 3’
**L8A/R7K**	SSIEFAKA	5’ GTT G**TA CGT A**AA CGT agc CtT GGC GAA CTC GAT GGA GGA GGT GGT CTT GAT GCG CTC CA 3’
**S1G/I3N/F5L/E4S(SIFE)**	GSNSLARL	5’ GTT G**TA CGT A**AA Ctg CAG CCT GGC cAA gct GtT GGA ccc GGT GGT CTT GAT GCG CTC CA 3’

*The restriction site of SnaBI used in cloning is shown in bold. Changes from the wild type sequence are shown in lower case letters.

**Fig 2:**
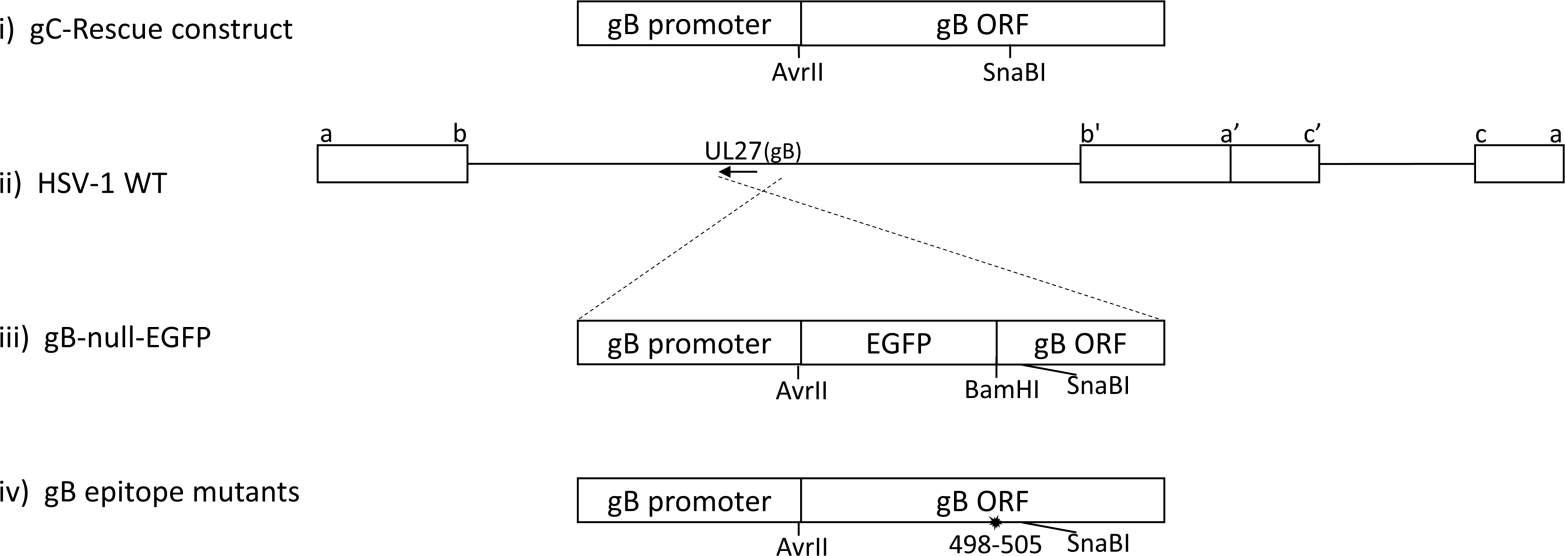
Construction of gB-null virus and of HSV-1 with gB_498-505_ mutations. Line i represents the parental plasmid used for derivation of the constructs in this study, detailed previously [47]. Line iii represents the replacement of the gB ORF with EGFP followed by the remaining part of the gB ORF from residue 509 to the end (gB ORF Back) that was developed to obtain a gB-null-EGFP virus. Line ii represents the HSV genome and the approximate coding position and direction of the gene for gB. Line iv represents the replacement gB genes and the site of the epitope mutations with respect to the SnaBI site used for derivation, as detailed in the text. AvrII and SnaBI are restriction sites used to clone the replacing region of gB.

**Fig 3:**
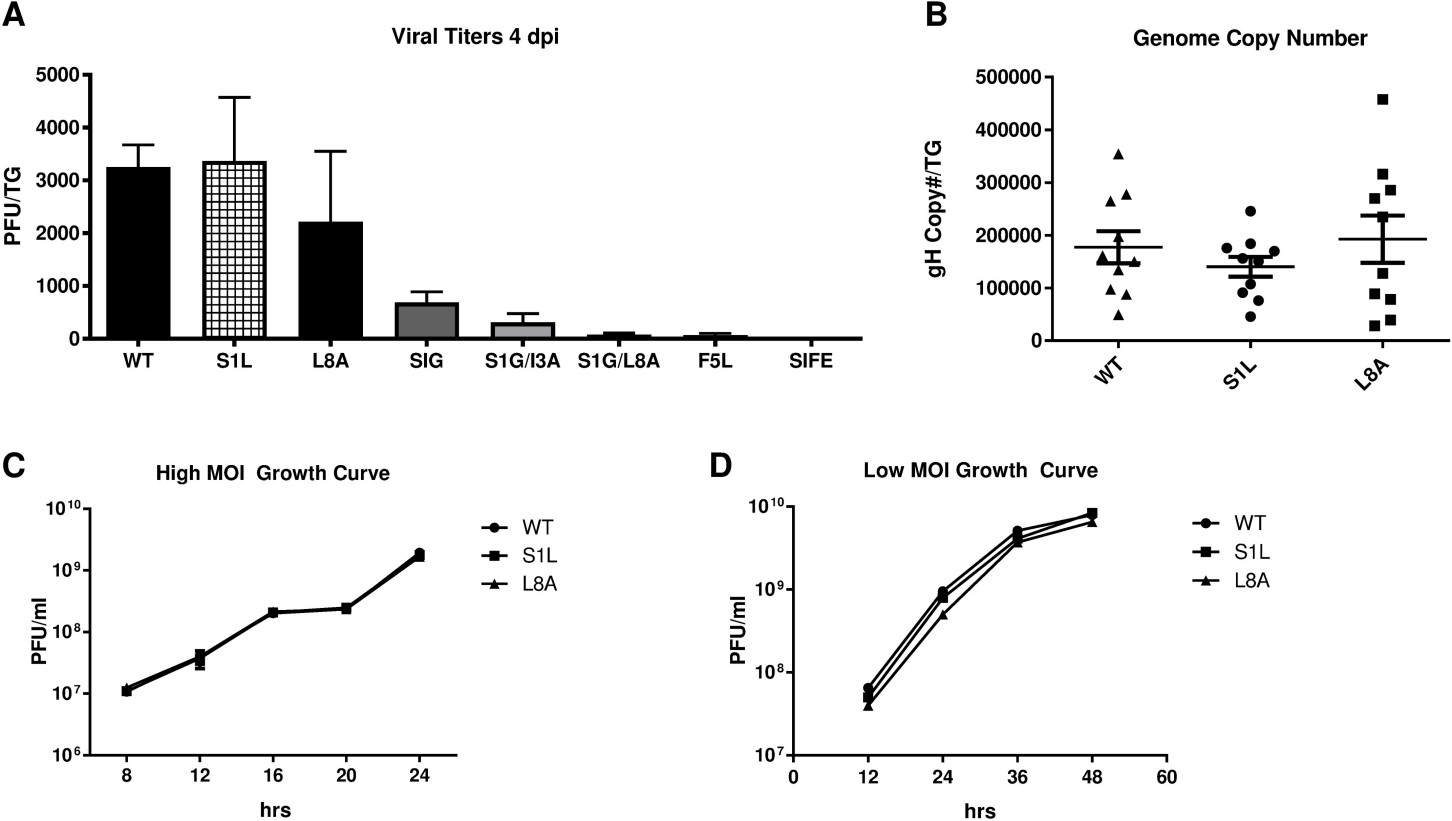
Growth of HSV gB mutants *in vitro* and *in vivo*. **(A)** Virus growth in the TG of B6 mice was determined at 4 days post ocular infection with 1x10^5^ PFU of either HSV-1 WT or HSV-1 containing the gB_498-505_ epitope mutants detailed in Table 1. TG were harvested and subjected to three freeze thaw cycles and infectious virus released into the supernatant was titrated on Vero cells. The graph represents the mean virus titer for each virus ± SEM of the mean (n = 5 mice). This is a representative of two separate studies with similar results. **(B)** Genome copy number determined by qPCR in the TG of mice infected with HSV-1 WT, S1L, or L8A following harvest at day 8 post ocular infection (n = 10). Values are representative of the total copies per TG. **(C,D)** Monolayer cultures of Vero cells were infected at a multiplicity of infection (MOI) of 10 PFU/cell (high MOI Growth Curve) or 0.01 PFU/cell (Low MOI Growth Curve) respectively with HSV-1 WT, S1L, or L8A. At the indicated hours post-infection, cells and supernatants were pooled, subjected to three freeze–thaw cycles and the viral titers were determined by plaque assay. The mean PFU/culture ± standard error of the means (SEM) is shown at each time.

HSV-1 recombinants containing each mutation were developed (Fig 2) by rescue of the growth of a gB-null-EGFP virus on gB-complementing Vero cells and all yielded virus that was able to form plaques on non-complementing cells. Recombinant HSV-1 with the following mutations in gB formed small plaques, indicating they were growth impaired: S1G, F5L, S1G/I3A, S1G/L8A, and S1G/I3N/F5L/E4S (Table 1). This was confirmed following ocular infection of B6 mice, where low viral replication was detected at 4 dpi within the TG of mice (Fig 3A). These impaired viruses were not studied further. However, HSV-1 S1L and L8A viruses could replicate to levels not significantly different from WT virus at 4 dpi in the TG (Fig 3A). They also establish equivalent latent genomic loads in the TG at 8 dpi (Fig 3B), and replicate to the levels of parental WT virus in both multi-step (infected at MOI of 0.01) and single step (infected at MOI of 10) growth curves in cultured Vero cells (Fig 3C and 3D). Since viral load and fitness in mice might influence CD8^+^ T cell hierarchy [34], we focused on these two HSV-1 mutants initially and then conducted detailed studies on HSV-1 S1L.

### HSV-1 lacking the gB_498-505_ epitope induces an equivalent TG CD8^+^ T cell response that is not directed to gB_498-505_

**Fig 4:**
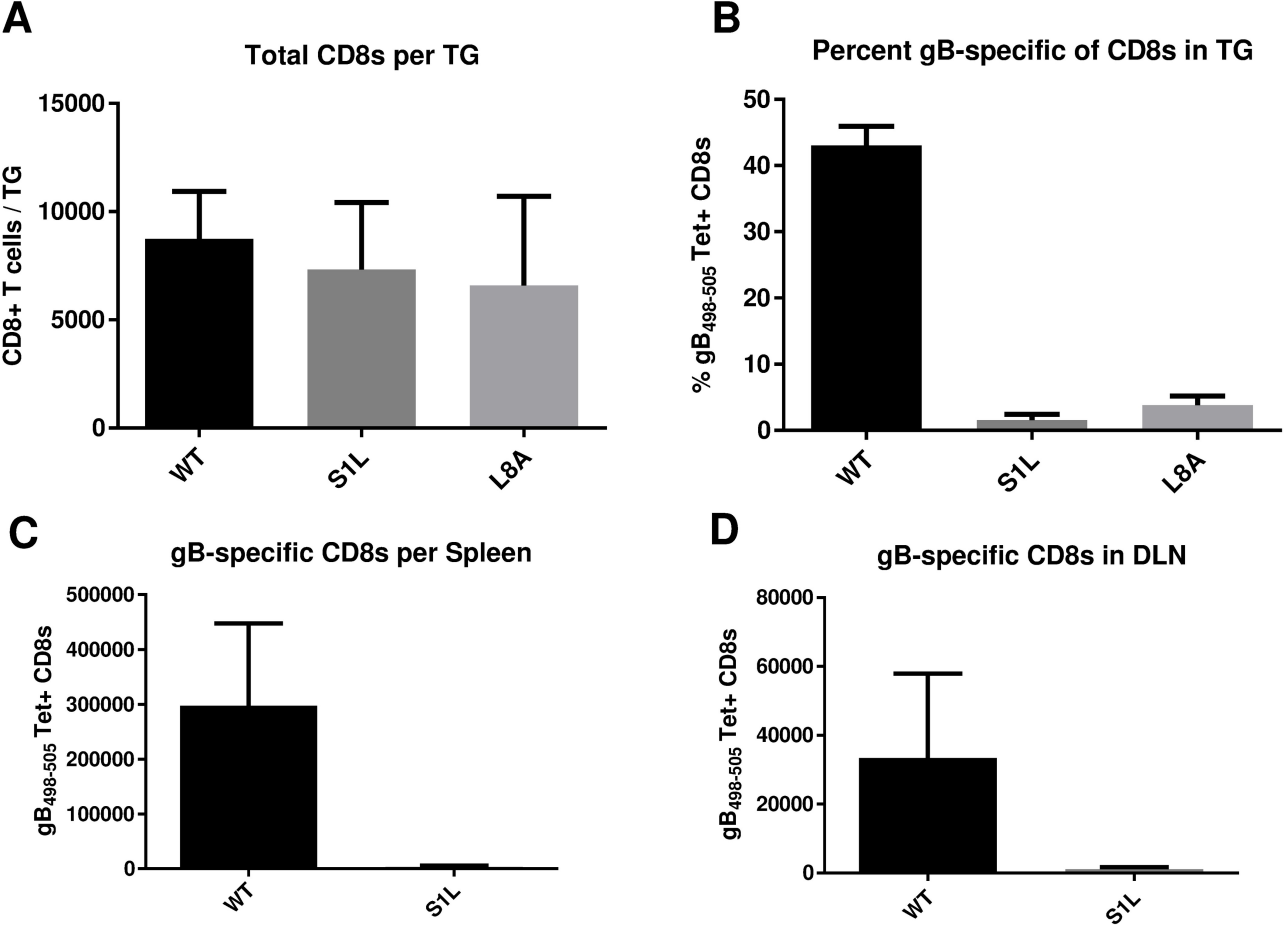
Acute CD8^+^ T cell infiltrates in the ganglia of mice after corneal infection with WT HSV-1 or recombinant HSV-1 containing gB_498-505_ mutations. Corneas of mice were infected with 1x10^5^ PFU/eye of HSV-1 WT, S1L, or L8A. At 8 dpi (peak CD8^+^ T cell infiltrate), the TG, spleen, or DLN were dissociated into single cell suspensions and surface stained with antibodies to CD45, CD3, CD8 and with MHC-I gB_498-505_ tetramer as detailed in Methods. Cells were analyzed by flow cytometry, and the data are presented as the mean +/- SEM (n = 5 mice, 10 TGs) of **(A)** absolute number of CD3^+^CD8^+^ T cells per TG, **(B)** the percent of gB_498-505_ tetramer positive CD8^+^ T cells in each TG, or **(C, D)** the total number of gB_498-505_ tetramer specific cells per spleen and local DLN. The experiment shown is representative of three additional experiments, all producing similar results. The absolute numbers of CD8^+^ T cells induced in the TG with each virus were not statistically different as shown by a one-way ANOVA followed by Tukey’s posttest (p = 0.58).

Following ocular infection of B6 mice, the peak CD8^+^ T cell infiltration in the TG occurs at 8 dpi and subsequently contracts to a low but persistent level that remains for the life of the host. The total CD8^+^ T cell infiltrates into TG of mice that received corneal infections with HSV-1 S1L and L8A were not statistically different from those induced by WT HSV-1 infection (Fig 4A). However, while approximately half of the CD8^+^ T cells infiltrating TG infected with WT HSV-1 [35] stained positively with gB_498-505_ H2-K^B^ tetramers, CD8^+^ T cells infiltrating TG infected with HSV-1 S1L and L8A showed extremely low numbers of gB_498-505_ tetramer positive cells (Fig 4B). The gB_498-505_ tetramer positive cells were also virtually undetectable in spleens and the local draining lymph nodes (DLN) of S1L infected mice (Fig 4C and 4D). This suggests that there is an HSV-specific CD8^+^ T cell response in the TG that compensates for the loss of the immunodominant gB-CD8 population, as seen previously[33].

**Fig 5:**
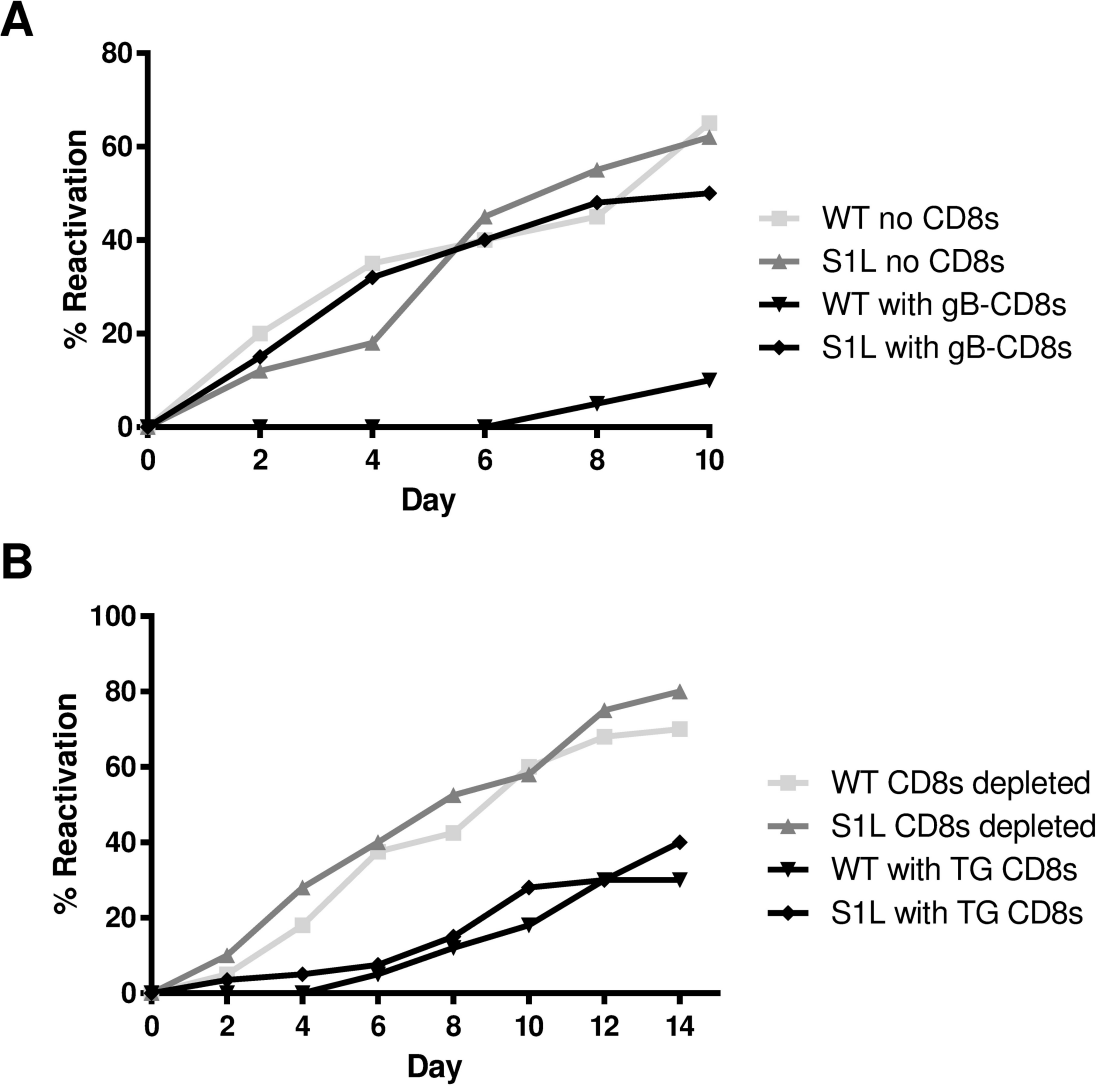
*Ex vivo* ganglionic reactivation of WT and S1L HSV-1. Corneas of B6 mice were infected with 1x10^5^ PFU of WT or S1L HSV-1. At 34 dpi latently infected TGs were dispersed with collagenase. **(A)** The TG cells were depleted at >95% of endogenous CD8^+^ T cells and distributed to wells of a 48-well tissue culture plate (0.2 TG equivalent/well) and cultured in culture medium containing IL-2 and with or without 2 x 10^4^ gB-CD8s added per well. Culture fluid samples were removed and replaced with fresh media every two days. The presence of infectious virus in culture fluid (indicating HSV-1 reactivation) was then determined by plaque assay. **(B)** TG cells were mock depleted or depleted of 95% of endogenous CD8^+^ T cells by treatment with anti-CD8α antibody and complement, then distributed to wells of a tissue culture plate and cultured as described in **A** above. (A & B) Data plotted as total percentage of wells that reactivated (showing infectious virus in culture supernatant) at the indicated time of culture. n = 10 TG per condition. Data for each experiment are representative of one of two repeats, but experiment-to-experiment variability in reactivation rates are routinely observed.

Failure of gB-CD8s to recognize the S1L mutation was further assessed by testing the ability of exogenous gB-CD8s to block HSV-1 S1L reactivation in *ex vivo* ganglionic explant cultures. TG of B6 mice harboring equivalent WT HSV-1 or S1L virus were excised at latency (34 dpi), dispersed with collagenase, depleted of endogenous CD8^+^ T cells and then plated in 1/5 TG cell equivalents per culture well, either alone or with 2x10^4^ gB-CD8s from a previously described T cell clone, all under conditions that maintain T cell viability [25]. In the absence of CD8^+^ T cells, approximately 50–60% of TG cultures showed HSV-1 reactivation and virus release into the media, with reactivation frequency that was not statistically different in TG infected with WT and S1L virus (Fig 5A). These data establish that HSV-1 S1L possesses robust ability to establish latency and reactivate in *ex vivo* cultures. However, in the presence of gB-CD8s, the reactivation of WT HSV was nearly abrogated, as expected from similar previous studies[19, 21]. In contrast, reactivation of S1L was not affected by addition of gB-CD8s, as a similar proportion of cultures reactivated with or without gB-CD8s (Fig 5A). However, the endogenous CD8^+^ T cells in TG that were latently infected with S1L HSV-1 were still able to inhibit S1L reactivation from latency as effectively as endogenous CD8^+^ T cells in TG latently infected with WT HSV-1 (Fig 5B). Taken together, these results indicate that S1L reactivation events do not appear to be recognized by gB-CD8s, but the endogenous S1L-induced CD8^+^ T cell response is equally effective as that of WT at reducing reactivation events.

**Fig 6:**
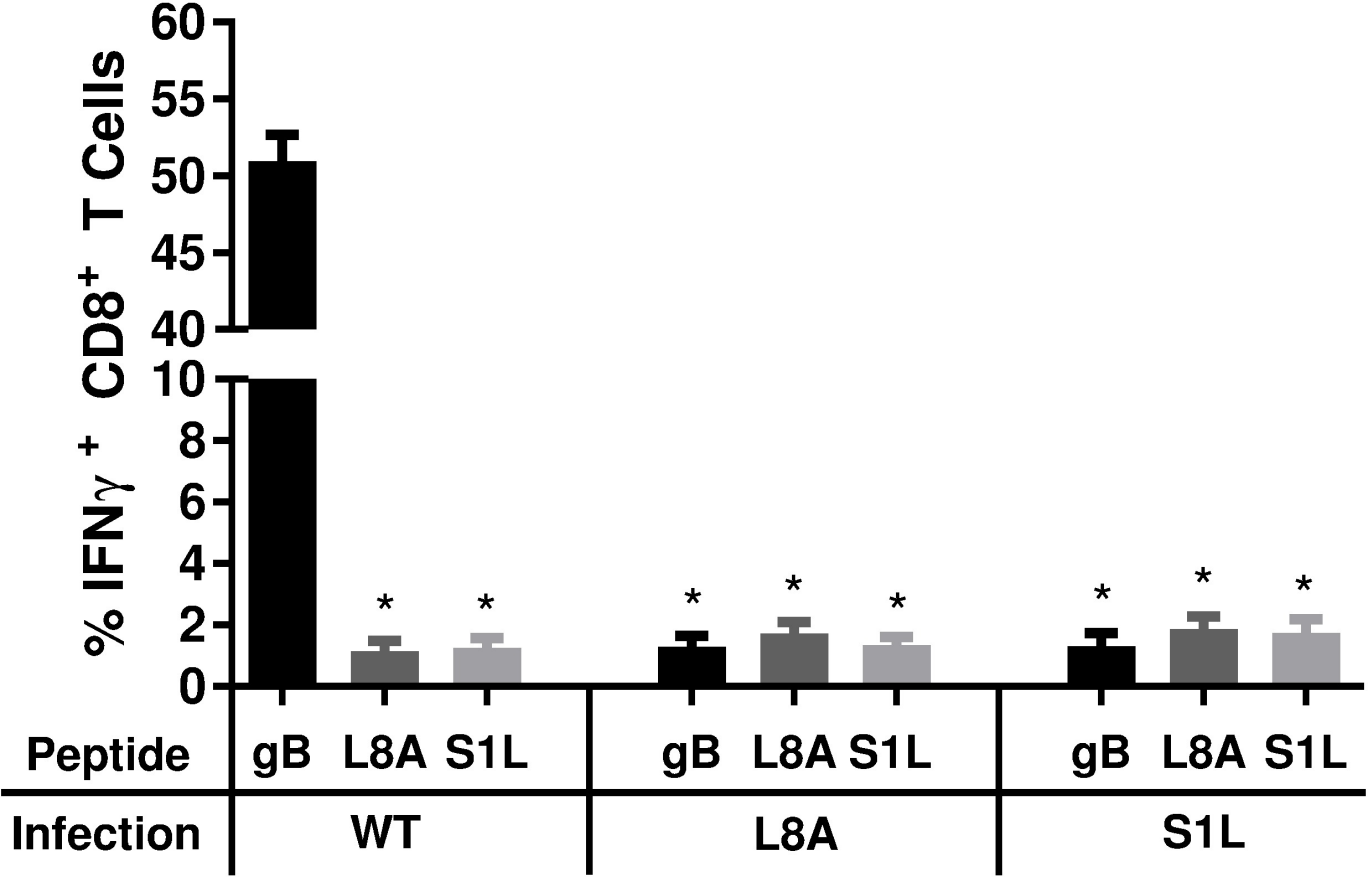
Stimulation of acute TG-resident CD8^+^ T cell populations with WT, SIL, or L8A gB peptides. B6 mice received corneal infections with HSV-1 expressing WT, S1L, or L8A gB. TG were obtained at 8 dpi, dispersed into single cell suspensions, and the endogenous CD8^+^ T cells were stimulated for 6 hours with B6WT3 fibroblasts pulsed individually with WT, S1L, or L8A gB_498-505_ peptides, in the presence of brefeldin A. Cells were surface stained for CD45 and CD8, followed by an intracellular stain for IFNγ. The data are represented as the mean percentage of CD8^+^ T cells that produced IFNγ +/- SEM (n = 5 mice per group). * represents significance of p<0.0001 for each group compared to gB peptide stimulation of wild-type infected control (first column) using one-way ANOVA with Dunnett’s multiple comparisons posttest.

Our previous work demonstrated that the vast majority of CD8^+^ T cells in WT HSV-1 acutely infected TG at 8 dpi are HSV-1 specific [27]. We considered several possible explanations for the compensated CD8^+^ T cell TG infiltrate observed here (Fig 4) in the absence of gB-CD8s. We assessed if a CD8^+^ T cell response developed to the mutated forms of the gB_498-505_ epitope. B6WT3 fibroblasts were pulsed with the S1L, L8A, or WT gB_498-505_ peptides and co-cultured with CD8^+^ T cells from TGs of S1L, L8A, or WT infected B6 mice taken at 8 dpi. The ability of CD8^+^ T cells to recognize these peptides was determined by staining CD8^+^ T cells for intracellular IFNγ production (Fig 6). Ganglionic CD8^+^ T cells induced by a WT HSV-1 infection responded robustly to stimulation with WT peptide-pulsed fibroblasts, but failed to respond to B6WT3 cells pulsed with S1L and L8A peptides. This suggested there is little or no cross-recognition of the mutant peptides by gB-CD8s. Furthermore, the compensated ganglionic CD8^+^ T cell populations induced by HSV-1 S1L or L8A infection failed to produce IFNγ when stimulated with fibroblasts pulsed with any of the 3 peptides (Fig 6). This strongly suggested that the compensation of the ganglionic CD8^+^ T cell response was not due to the development of CD8^+^ T cells directed to the mutated gB peptides.

### A compensatory response to HSV-1 subdominant epitopes in S1L-infected TG

**Fig 7:**
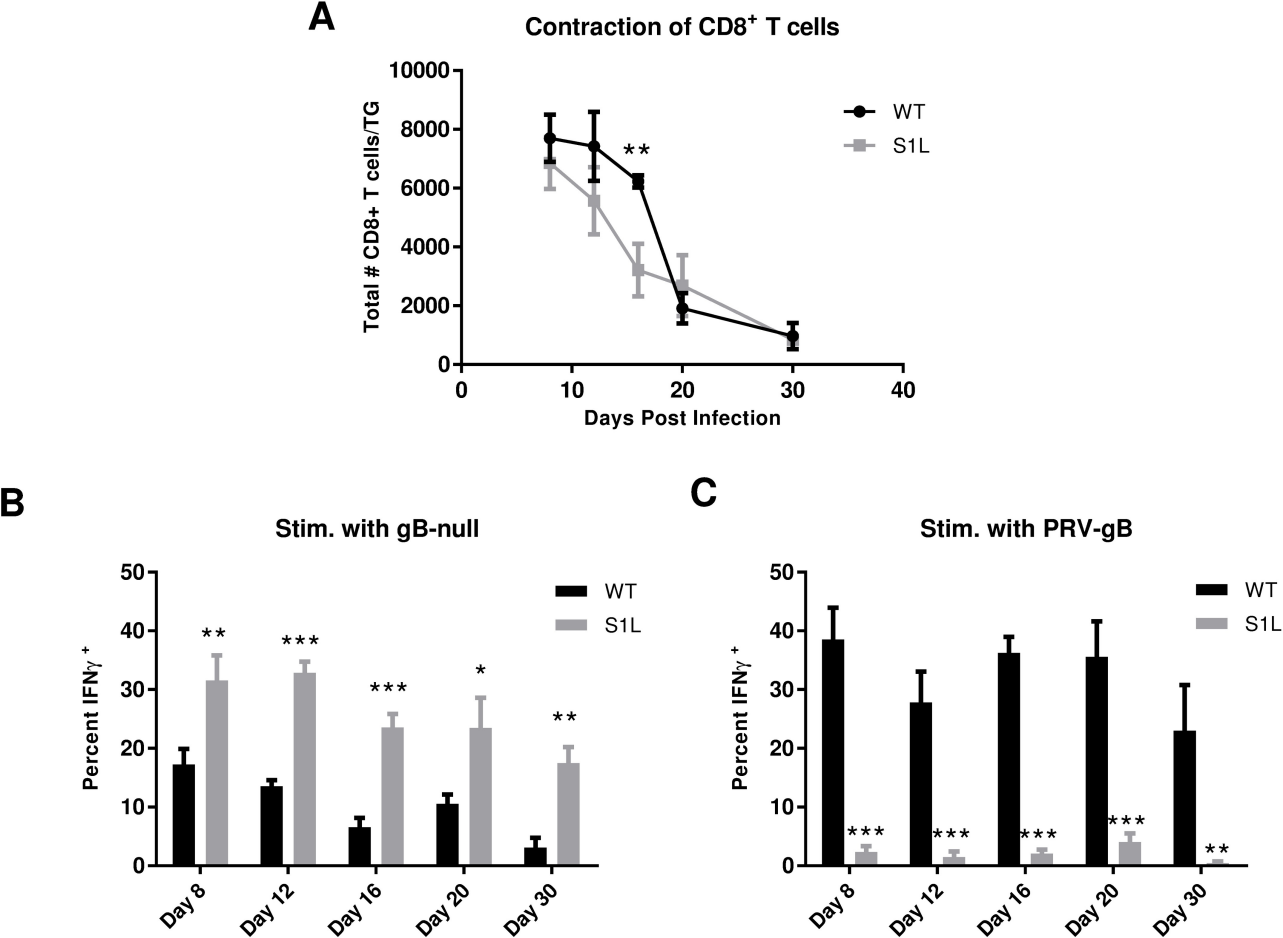
The CD8^+^ T cell population in S1L infected TG contract more rapidly and contain a higher frequency of active non-gB-CD8s. B6 mice received corneal infections with either WT or the S1L mutant at 1x10^5^ PFU/cornea. TGs were harvested at 8, 12, 16, 20, or 30 dpi and: **(A)** stained for CD45, CD3, and CD8, analyzed by flow cytometry, and data recorded as the mean number of CD8^+^ T cells/TG; or stimulated for 6 hrs with **(B)** HSV-1 gB-null-EGFP infected or **(C)** PRV-gB infected B6WT3 fibroblasts in the presence of Brefeldin A. The cells were then stained for surface CD45, CD3, and CD8 and for intracellular IFNγ. Data in B and C are presented as the mean ± SEM frequency of IFNγ^+^ CD8^+^ T cells in each TG as a fraction of total CD8^+^ T cells. * p<0.05, ** p<0.01, ***p<0.001 based on a t-test comparison at each time.

We next addressed the possibility that the compensated CD8^+^ T cell responses to S1L contained an expansion of CD8s directed to other HSV-1 epitopes. CD8^+^ T cells obtained from TG of mice infected with either WT or S1L HSV-1 were assessed both for total ganglionic CD8^+^ T cell infiltrates (Fig 7A) and for their ability to be stimulated by infected fibroblasts at different times post-infection (Fig 7B and 7C). The total ganglionic CD8^+^ T cell infiltrate for both WT and S1L showed a similar characteristic peak at 8 dpi and a subsequent contraction by 30 dpi (Fig 7A). However, at 16 dpi S1L infected TGs had a significantly smaller CD8^+^ T cell infiltrate, suggesting a more rapid CD8^+^ T cell contraction occurred.

To assess the response to non-gB_498-505_ HSV-1 epitopes, TG from WT or S1L infected mice at different times post infection were dispersed, stimulated for 6 hrs with B6WT3 fibroblasts infected with HSV-1 gB-null-EGFP virus, and intracellular IFNγ in CD3^+^CD8^+^ T cell populations was measured by flow cytometry (Fig 7B). We found a significantly higher frequency of IFNγ^+^ CD8^+^ T cells in S1L-infected TG compared to WT infected TG following stimulation with UV-irradiated B6WT3 cells infected with HSV-1 lacking gB. At the acute stage (8 dpi), the frequency of S1L stimulated CD8s was approximately twice that of the WT-stimulated CD8^+^ T cells. Intriguingly, in a WT infection, the fraction of ganglionic non-gB-CD8s responding to antigen drops over time. These data are consistent with our observation that in WT infected TG, CD8^+^ T cells specific for subdominant HSV-1 epitopes become functionally compromised by 30 dpi [30, 31]. However, we found that while non-gB_498-505_ CD8^+^ T cells in an S1L infection also dropped as latency was established, over 4x more non-gB-CD8s responded to infected target cells by producing IFNγ in this assay. This is more than can be explained by a simple doubling of stimulated cells that would be expected to fill the gB-CD8 compartment. This suggests that these non-gB-CD8s still target HSV-1 epitopes and are more functional in TG infected with HSV-1 S1L.

To separately assess CD8^+^ T cell responses to the gB immunodominant epitope in a similar assay, we stimulated CD8^+^ T cells obtained from S1L and WT latently infected TG for 6 hrs with B6WT3 cells infected with a recombinant pseudorabies virus (PRV) that expresses the HSV gB residues 494–509 containing the HSV immunodominant peptide (SSIEFARL) under control of the CMV promoter (PRV-gB) and measured intracellular IFNγ (Fig 7C). CD8^+^ T cells in S1L infected TG responded minimally to stimulation with the PRV-gB_494-509_ virus infected cells whereas CD8^+^ T cells in WT HSV-1 infected TG showed a robust response that maintained high levels throughout establishment of latency. These data agree with our previous finding that CD8^+^ T cells specific for the immunodominant gB_498-505_ epitope remain highly functional in TG latently infected with WT HSV-1 [30, 31]. They also demonstrate that CD8^+^ T cells in S1L-infected TG do not cross react to any presented PRV MHC-I epitopes in this assay. These results indicate that the compensatory response to S1L in the ganglia appears to reflect an increased number of CD8^+^ T cells directed to HSV epitopes other than gB_498-505_, and that these CD8^+^ T appear more functional in S1L infected TG compared to those in TG infected with WT HSV-1.

### Efficient TG-retention of gB_498-505_ specific CD8^+^ T cells requires antigen

**Fig 8:**
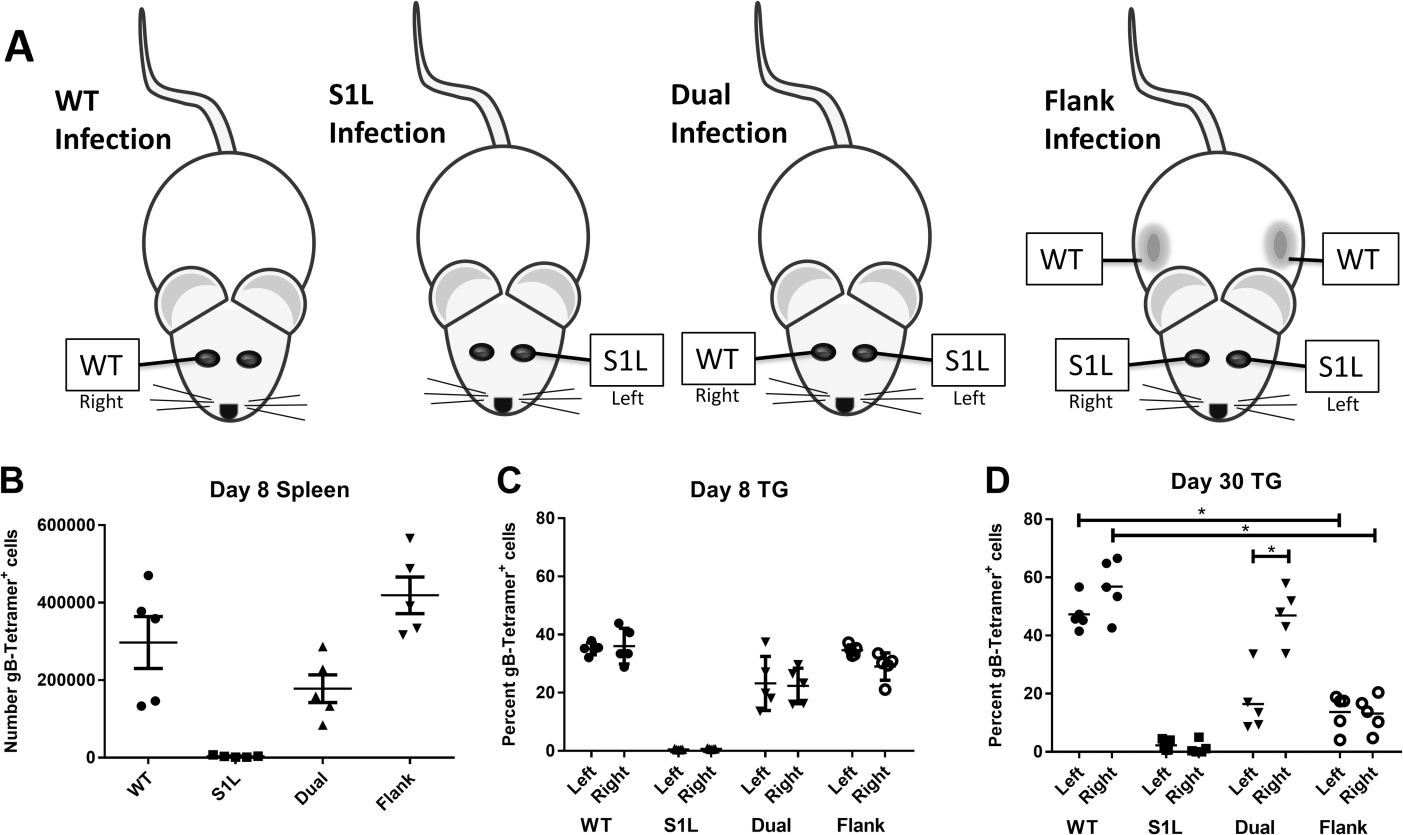
gB-CD8^+^ T cell retention in the HSV-1 latently infected ganglia is dependent on antigen expression. **(A)** Representation of infection models in which mice received unilateral corneal infections with WT (WT only) or S1L (S1L only) HSV-1, bilateral infections with WT on one cornea and S1L on the other cornea (dual infection); or bilateral corneal infection with S1L and flank infection with WT HSV-1. All corneal infections were with 1x10^5^ PFU/scarified cornea, and flank infections were with 1x10^6^ PFU on a scarified flank. At 8 or 30 dpi, TG and spleen suspensions were analyzed by flow cytometry for CD45, CD3, CD8, and gB-tetramer. **(B)** Total number of gB-tetramer^+^ cells/spleen. **(C and D)** Frequency of CD3^+^CD8^+^ T cells in the TG that are gB-tetramer^+^. * Statistical significance by one-way ANOVA with p<0.01.

It was previously shown that activated, exogenously introduced non-HSV-specific OT-I CD8^+^ T cells could enter the TG during acute infections, but were not retained in the TG over time, presumably due to lack of cognate antigen recognition within the tissue [35]. The availability of an HSV-1 lacking the immunodominant peptide allowed us to assess the necessity of HSV-1 antigen presence in retaining ganglionic CD8^+^ T cell populations *in vivo*. We performed simultaneous corneal infections with the S1L virus lacking the immunodominant gB_498-505_ epitope in conjunction with ocular or flank infections with WT virus (Fig 8A). These infection models were designed to induce a systemic CD8^+^ T cell response to the gB_498-505_ epitope and observe the retention of that response under conditions where the epitope was or was not expressed in the TG. As expected, mice receiving corneal infections of either WT or S1L virus developed acute systemic and TG CD8^+^ T cell responses that contained or lacked the gB_498-505_ specific CD8^+^ T cell populations, respectively (Fig 8B and 8C). When a gB_498-505_ specific CD8^+^ T cell response was primed in S1L ocular infected mice by coinfection with WT HSV-1, either in the other eye or by flank infection, we noted that the infiltrate of the S1L infected TG contained a CD8^+^ T cell response to gB_498-505._ Indeed, the CD8^+^ T cell infiltration of the S1L and WT infected TG were quite similar at 8 dpi, with equivalent levels of both gB-CD8s and non-gB-CD8s. This data fits with the previously reported observation that acute ganglionic infection draws most or all activated CD8^+^ T cell populations into the ganglia [35]. However, a different pattern emerged by 30 dpi at HSV latency. In WT latently infected TG, irrespective of whether or not they received simultaneous corneal infection with S1L virus, an approximate 50:50 proportion of gB-CD8 to non-gB-CD8 T cells was retained during latency (Fig 8D). However, in S1L latently infected mice that were co-infected with WT virus ocularly or by flank infection, the gB-CD8 populations were greatly reduced by 30 dpi in the S1L infected ganglia. These results strongly support the conclusion that while most activated CD8^+^ T cell populations are able to infiltrate the ganglia at acute stages of infection, the maintenance of ganglia-resident HSV-specific CD8^+^ T cell populations requires antigen expression within the TG.

### Defining the antigenic repertoire of the CD8^+^ T cell response to HSV S1L

**Fig 9:**
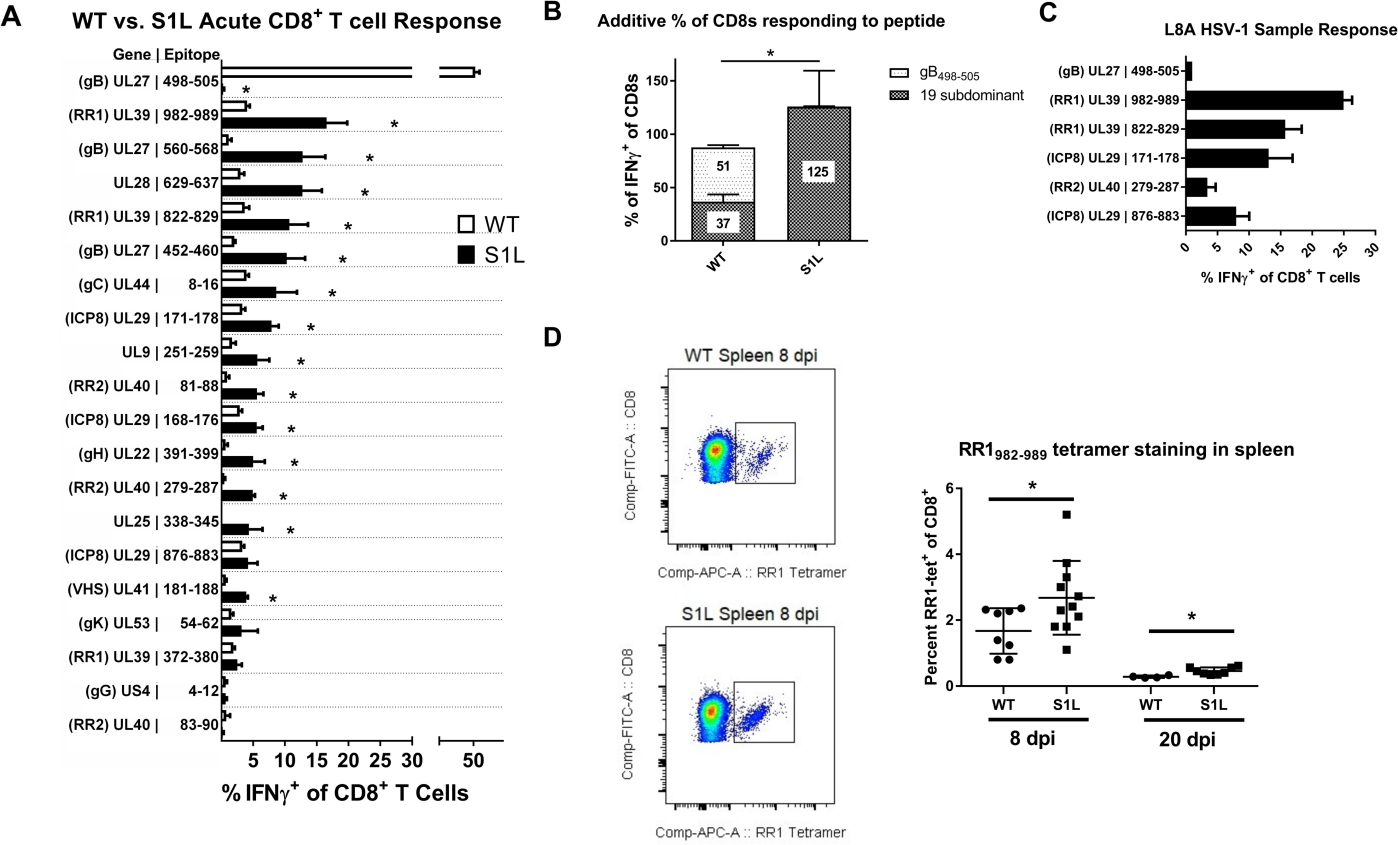
Subdominant HSV-1 epitopes expand to accommodate the loss of the immunodominant gB_498-505_ epitope during acute infiltration into the TG. B6 mice received corneal infections with **(A, B, D)** HSV-1 WT, S1L, or **(C)** L8A. TG were excised at 8 dpi, dispersed into single cell suspensions, stimulated for 6 hrs in the presence of Brefeldin A with B6WT3 cells pulsed with peptides corresponding to known HSV-specific CD8^+^ T cell epitopes, stained for surface CD45, CD8, and intracellular IFNγ. **(A)** The graph shows the percent of the total CD8^+^ T cell population staining for intracellular IFNγ by flow cytometry. The bars represent the mean ± SEM frequency of CD8^+^ T cells producing IFNγ in response to each epitope. **(B)** Total fraction of gB_498-505_ or non-gB-CD8s responding to peptide stimulations as seen in (A). N = 3–8 TG equivalents per peptide. **(C)** A fraction of the peptide library was analyzed as in (A), but in mice infected with HSV-1 L8A. **(D)** Spleens were also excised at 8 and 20 dpi and examined for RR1_982-989_ tetramer positive cells. Shown is example of tetramer staining, and a graph depicting the total fraction of splenic CD8+ T cells that stained positive for tetramer. A t-test was performed for each matched pair of responding CD8^+^ T cells, and * denotes p<0.05.

Virtually the entire CD8 antigenic repertoire to WT HSV-1 in B6 mice was recently defined [27]. This gave us the opportunity to examine the specific nature of the compensation to HSV-1 subdominant epitopes. We utilized the known HSV-1 subdominant epitope library to determine the size of each subdominant-epitope population that responds to peptide within the TG. HSV-1 induced CD8^+^ T cells infiltrating the ganglia at 8 dpi were evaluated for their ability to produce cytokines following stimulation with B6WT3 cells pulsed with each known epitope peptide. For WT, just over half of the CD8^+^ T cells in the TG of infected mice at 8 dpi responded to the immunodominant gB_498-505_ epitope, while the remaining CD8^+^ T cells responded at much lower levels to the 19 subdominant epitopes reported previously, with the addition of one minor epitope that is discussed below [27]. In contrast, CD8^+^ T cells infiltrating S1L infected TG showed no reactivity to the gB_498-505_ epitope, but showed a statistically significant increase in responses to most of the tested subdominant epitopes (Fig 9A). The response to a few subdominant epitopes remained statistically unaffected in the HSV S1L induced CD8^+^ T cell population, though an overall trend of increased levels was evident. Epitopes that featured prominently in the altered dominance hierarchy induced by HSV-1 S1L included RR1 (ribonucleotide reductase large subunit 1, UL39), with T cell epitope frequencies being the most abundant (RR1_982-989_) and fourth most abundant (RR1_822-829_) in the altered hierarchy. Interestingly, gB_560-567_ rose to position two in the hierarchy. Addition of the fractions responding to each peptide indicated that most of the CD8^+^ T cells were accounted for, with the fraction responding to each epitope adding to a total of 125.6 ^+^/-33.8 percent(Fig 9B). This strongly suggests that the compensation was not a result of the development of significant CD8^+^ T cell populations to new epitopes, although we cannot exclude the possibility that minor populations directed to previously unreported epitopes were present in HSV-1 S1L infected mice. We also evaluated a subgroup of five of the specific epitopes for recognition by the HSV-1 L8A induced ganglionic CD8^+^ T cell response, and the results suggested this virus induced a similar compensatory response to that of S1L (Fig 9C). Finally, it was reported by Stock, et al [33]that the systemic response to the only known epitope at that time (RR1_822-829_) did not expand systemically; In contrast, we found that RR1_982-989_-specific CD8^+^ T cells, defined by tetramer, were expanded at both acute and late times (Fig 9D). These results indicate that the compensatory CD8^+^ T cell response to HSV-1 lacking the immunodominant gB epitope at 8 dpi is due to increased CD8^+^ T cell populations directed to the other tested subdominant epitopes.

**Fig 10:**
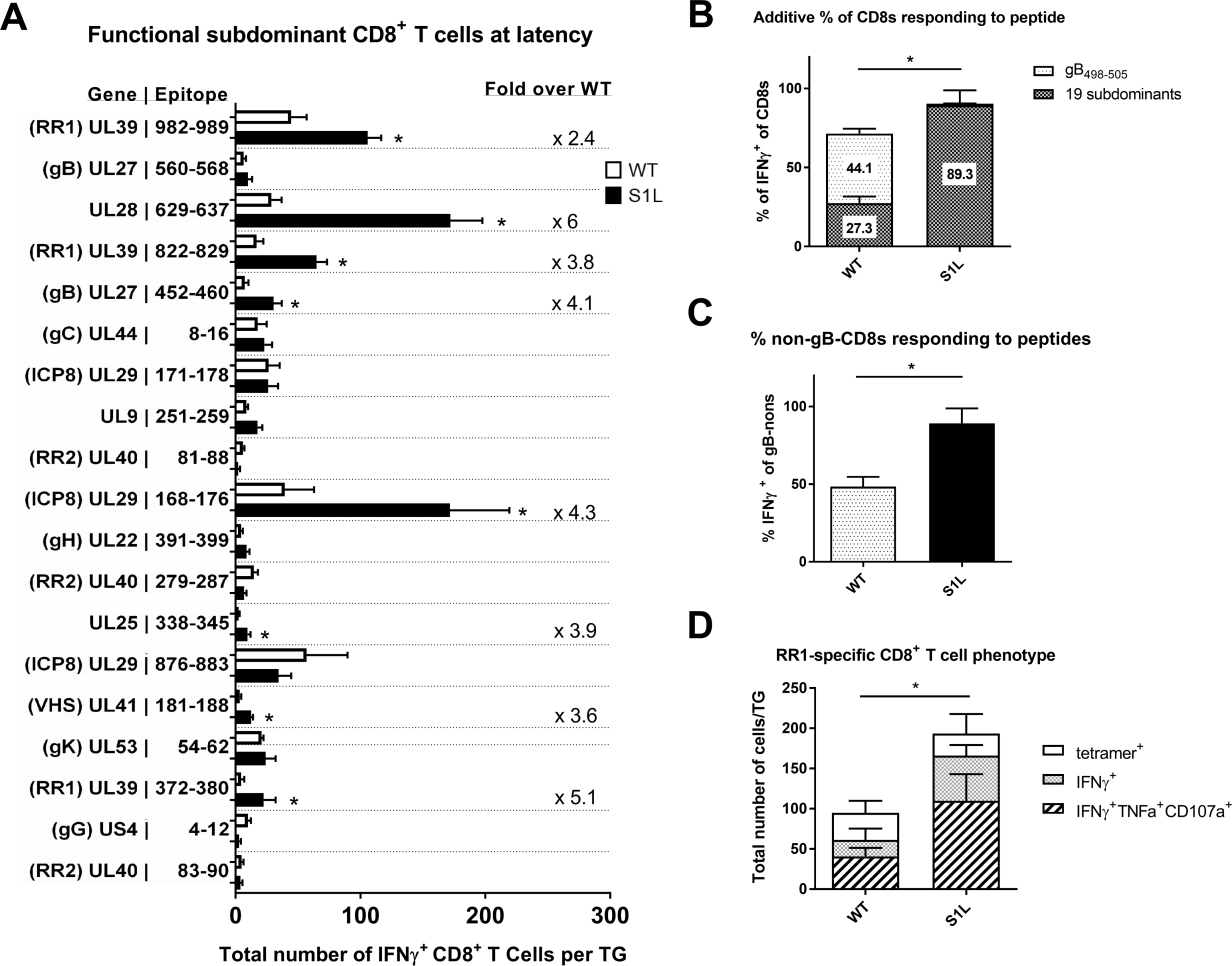
Certain subdominant HSV-1 epitopes become more functional and arise to codominance in TG during HSV-1 S1L latency. Studies were as detailed in Fig 9, except that TGs were harvested from infected mice at 30–33 dpi. **(A)** The order of epitopes is identical to those shown in Fig 9. For clarity, only the non-gB_498-505_ responses are shown in A, but the total percentages are displayed in (B). The bars represent the total number of CD8^+^ T cells producing IFNγ^+^ in response to peptide stimulation, and error bars represent SEM. For significantly different populations, the average fold change increase in population over wild-type is shown. **(B)** Total fraction of gB_498-505_ or non-gB-CD8s depicted in figure (A) that are responding to peptide stimulations. **(C)** Total fraction of the non-gB_498-505_ specific CD8+ T cells depicted in figure (A) that make IFNγ after peptide stimulation. **(D)** 30 dpi TGs suspensions were stained with tetramers specific to RR1-specific subdominant CD8+ T cell populations (tetramers for RR1 982–989 and 822–829). Shown are the total number of each RR1-specific CD8+ T cell population per TG. The total number of single functional (IFNγ^+^) or multifunctional (IFNγ^+^TNFα^+^CD107a^+^) CD8^+^ T cells after stimulation with a combination RR1 982–989 and 822–829 peptides is also shown. N = 3–7 TG equivalents per group. A t-test was performed for each matched pair of responding CD8^+^ T cells, and a * denotes p<0.05.

Intriguingly, a quite different pattern emerged at 30 dpi, when HSV-1 is considered to be latent. We show the total average numbers of non-gB CD8^+^ T cells per latently infected ganglion that produce IFNγ^+^ in response to peptide presentation (Fig 10A). In a WT infection, the ganglionic CD8^+^ T cells to gB_498-505_ still responded to stimulation with peptide pulsed cells, and accounted for nearly half of the total CD8^+^ T cells detected in the ganglia (Fig 10B). However, the combined non-gB-CD8 populations in WT HSV-1 infected TG showed a much poorer response, with a total of only 27.3% of the subdominant CD8s producing IFNγ when stimulated with each of the subdominant peptides on B6WT3 cells. This data fits well with earlier reports indicating that a significant fraction of the non-gB-CD8s in the WT latently infected ganglia are not able to respond efficiently to antigen[30]. In contrast, the landscape of epitopes to which S1L induced CD8^+^ T cells can respond was changed dramatically, with 3–4 epitopes rising to prominence or co-dominance (Fig 10A). The most prominent were to UL28_629-637_(DNA packaging terminase), ICP8_168-176_, and the two large subunit ribonucleotide reductase epitopes; RR1_982-989_ and RR1_822-829_. This contrasts to the sub-dominant response hierarchy against WT HSV-1, which changed little over time. Secondly, when the CD8^+^ T cells from S1L latently infected ganglia that responded to peptide were totaled, 89.3 ± 16.5% of the CD8^+^ T cells were functionally able to produce IFNγ upon stimulation (Fig 10B). This does not simply reflect enrichment due to the lack of a gB_498-505_ response since the proportion of functional non-gB_498-505_ cells in this assay were significantly higher (Fig 10C). This is also in line with the data presented in Fig 7B showing that loss of the immunodominant epitope results in a functional increase in non-gB-CD8s at latency.

Comparing the total fraction of a CD8^+^ T cell population present by tetramer, to that identified by IFNγ production after peptide stimulation should tell us what percentage of that specific population is functional. We could not obtain working tetramers for most CD8 populations described here, but we were able to further characterize the functionality of RR1_982-989_ and RR1_822-829_ CD8^+^ T cell populations. This was done both by tetramer staining, and by determining multifunctionality (ability to produce multiple cytokines) following a peptide stimulation (Fig 10D). In a wild-type infected TG at latency, about 95 CD8^+^ T cells stained positive for these tetramers, with about 64% of these cells producing IFNγ, and 43% exhibiting multifunctionality (IFNγ^+^, TNFα^+^, and CD107a^+^) after stimulation. In contrast, an average of about 204 CD8^+^ T cells were tetramer positive in an S1L infected TG, with 84% capable of producing IFNγ, and 54% being multifunctional. This increase in functionality was statistically significant. Taken together, these results suggest that the presence of a strongly immunodominant CD8^+^ T cell population in latently infected ganglia adversely affects the functionality of those reactive to subdominant epitopes.

## Discussion

Given the general propensity of CD8^+^ T cells to recognize only a limited and highly restricted array of the potential epitope repertoire of a pathogen and develop a hierarchical response to them, it is important to understand how the presence or absence of one epitope, particularly an immunodominant epitope, influences the functionality and hierarchy of the CD8^+^ T cell response to others for a specific pathogen. Recent studies have revealed that HSV-1 specific CD8^+^ T cell responses developing to prior HSV infections in humans are highly limited, with only select proteins arising to dominance or co-dominance[36]. The unusually strong immunodominance to gB_498-505_ in the B6 mouse model of HSV-1 infection and the known full CD8^+^ T cell hierarchy makes it a highly suitable and manipulatable model to address the issue of how loss of one epitope affects the remaining response. Previously, the nature of CD8 compensation for loss of immunodominance could not be more precisely defined for HSV, because the HSV-specific CD8^+^ T cell repertoire in B6 has only recently been described [27]. That study demonstrated that the entire HSV-1 CD8^+^ T cell repertoire generated in the spleen was represented and not significantly modified in the TG, and that the vast majority of CD8^+^ T cells in the acutely infected TG at 8 dpi were HSV-specific [35].

Prior to this work, only one study had addressed the question of immunodominance loss in HSV-1, wherein B6 mouse skin infection with the HSV-1 L8A point mutation in the gB_498-505_ epitope was shown to induce a normal-sized expansion of HSV-specific CD8^+^ T cells in the spleen that lacked any specificity for the immunodominant gB_498-505_ epitope [33]. However, the authors concluded that the compensation reflected an additional response to previously unrecognized cryptic epitopes, based largely on the observation that CD8^+^ T cells to the one known subdominant HSV-1 epitope defined at that time, RR1_822-829_, were not increased in frequency of recognition by the compensated response. This finding is similar to a study by Holtappels et. al with murine cytomegalovirus (MCMV), wherein they demonstrated that deleting two MCMV immunodominant CD8^+^ T cell epitopes resulted in an altered immune response that was still protective, but contained small changes to known subdominant CD8^+^ T cells to known epitopes, with one specific epitope identified as rising in the absence of these deletions [37]. Similarly, Kotturi et. al deleted several immunodominant epitopes within Lymphocytic Choriomeningitis virus (LCMV), and show the altered immune response contained only limited increases in the subdominant CD8^+^ T cell hierarchy [38]. In our current study, we have found a somewhat different result in HSV-1 latently infected ganglia. Here we define the complete nature of the compensatory response in the TG and show a broad increase in the numbers and function of subdominant CD8 populations. Furthermore, assessment of the splenic response for one epitope indicates that at least part of the subdominant compensation occurs systemically in our model (Fig 9).

Of the several recombinant viruses developed to lack the gB_498-505_ epitope, two retained WT levels of pathogenicity in the mouse model. We chose S1L for most of the detailed studies, since L8A trended toward a marginally reduced ganglionic infiltrate in the TG (Fig 4) although differences were not significant. Reduced virus replication in the eye or TG may affect the level of antigen available for CD8^+^ T cell priming, recruitment and/or retention, as has been observed in the LCMV model [39]. S1L not only established latency with the same genomic loads as WT HSV-1, but also reactivated to the same efficiency in the absence of T cells. Both S1L and L8A viruses induced total ganglionic CD8^+^ T cell responses numerically identical to WT, despite the fact that the ~50% gB_498-505_ specific response was absent. Importantly, we show that neither S1L nor L8A virus induced a CD8^+^ T cell response to the native or mutated gB_498-505_ epitope, as demonstrated by priming and peptide stimulation assays (Fig 6). The lack of response to the S1L modified gB_498-505_ epitope was also established by demonstrating that (i) CD8^+^ T cells from mice infected with WT HSV failed to respond to the S1L modified gB peptide: (ii) CD8^+^ T cells in TG harboring the S1L virus showed negligible binding to tetramers containing the native gB_498-505_ epitope; and (iii) gB_498-505_-specific CD8^+^ T cells that effectively prevented reactivation of the parental wild-type HSV-1 strain had no effect in reducing reactivation of S1L from latently infected TG in *ex vivo* cultures. The epitope-specific nature of the reactivation blockade by gB-CD8s (Fig 5) also provides strong evidence that MHC-I presentation and cognate CD8^+^ T cell recognition are absolutely required to stop viral exit from latency in this model. This is consistent with our previous finding that HSV-1 specific CD8^+^ T cells from C57BL/6 mice can block HSV-1 reactivation in latently infected TG of C57BL/6, but not BALB/c mice [19], and contrary to a recent study suggesting that CD8^+^ T cells do not control HSV-1 reactivation events[40].

The nature of the compensated CD8^+^ T cell response in the acute infected TG to an immunodominant epitope-mutated HSV-1 reported here appears to be predominantly or entirely due to CD8s specific to other HSV-1 epitopes (Fig 7B). The compensatory increase of subdominant CD8^+^ T cell responses to epitope deleted viruses has been seen in other viruses, for example, following infection with an influenza virus that lacked an immunodominant epitope [41]. However, CD8^+^ T cell compensation was not observed following infection with epitope deleted LCMV, where the CD8^+^ T cell response was numerically reduced and delayed [42,43]. As noted by Stock et al [33] it is not clear if this difference in the ability to compensate for the loss of an immunodominant epitope reflects the difference between a local infection, like influenza and HSV-1, and a systemic infection like LCMV. Our studies using B6WT3 cells pulsed with each of the 19 known subdominant CD8^+^ T cell epitopes to stimulate IFNγ expression in CD8^+^ T cells indicate that most subdominant CD8 populations had an increased size to compensate for the loss of an immunodominant epitope. The added total CD8^+^ T cell response accounts for all (125.6% ± 33.8%) CD8^+^ T cells in S1L acutely infected TG, not only confirming that they are still HSV-1 specific, but suggesting negligible induction of CD8^+^ T cells specific for new cryptic HSV-1 epitopes. One possible exception to this is UL25_338-345_, which was a low frequency epitope identified, but not reported, in our previous study[27]. This CD8^+^ T cell population was identified as a small response (~0.2% of the total) in WT HSV-1 infections in our initial screen, but not in the subsequent repeat, suggesting a possible low frequency response in WT HSV-1. In an S1L infection, however, this epitope represented ~4% of the total acute TG infiltrate. We interpret this finding as an expansion of an existing low-frequency epitope-specific CD8^+^ T cell response. While we cannot exclude the possibility that S1L induced additional CD8^+^ T cell populations arising to new or cryptic epitopes, these would necessarily be small populations that would be difficult to identify via peptide library screening.

The change in the acute dominance hierarchy in the absence of the immunodominant gB_498-505_ epitope was not predictable. Compared to WT, the frequency of acute CD8^+^ T cells in the TG reactive to some epitopes such as UL39_982-989_ increased 4-fold, while the response to others such as UL29_876-883_ were only modestly increased. The response to others such as US4_4-12_ showed little change compared to WT. However, 14 of the 19 subdominant CD8^+^ T cell populations in the S1L infected ganglia showed a significant increase (Fig 9). The reason for this differential rise of epitope-specific CD8^+^ T cell responses in the absence of the immunodominant epitope is not clear. Priming of the initial CD8^+^ T cell hierarchy as well as expansion of memory subsets are very dependent on the context of naïve T-cell-APC interactions, and T cells directly compete for antigen on APCs very early in this process[44–46]. CD8^+^ T cell population hierarchies also depend on naïve precursor frequency as well as peptide processing and MHC binding strength[38]. Selection in our model is not completely attributable to MHC binding capacity, since epitopes that changed little in the dominance hierarchy such as UL29_876-883_ and UL40_83-90_ were previously reported [27] to have higher MHC binding capacity (IC_50_ = 6.2 nM and 5.4 nM, respectively). Epitopes such as gB_560_ that rose substantially in the S1L dominance hierarchy had a much lower MHC binding capacity (IC_50_ = 9206 nM). The naïve precursor frequencies for the full HSV-specific response in our model has not yet been fully explored, but is outside the scope of this study. The kinetics of viral protein production are also likely to influence CD8^+^ T cell hierarchy, since a majority (~80%) of the epitopes recognized by CD8^+^ T cells in B6 mice are encoded by viral early (β) and leaky late (γ1) genes. This would be consistent with our previous finding that expressing gB as a true late (γ2) gene greatly reduced gB_498-505_ immunodominance [47]. Other studies using MCMV, Vaccinia, and Epstein-Barr virus epitopes also suggest that the context and kinetics of viral antigen expression contribute heavily to CD8 immunodominance, with the trend of stronger and earlier expression and presentation being important for the development of immunodominant CD8 populations[48–51]. However, we note differential rise to dominance of epitopes on the same viral protein in TG infected with S1L, suggesting that the level of expression of a particular viral gene product cannot by itself explain changes in immunodominance.

In the LCMV model it has been observed that when the virus is cleared acutely, the lasting memory CD8^+^ T cell hierarchy is maintained at similar proportions, but when a chronic strain is used, the hierarchy changes over time[52]. It is thus intriguing that the hierarchy at latency in the S1L infected TG is quite different from the hierarchy at acute infection. With our dual eye infection or flank infection models, we show that gB-CD8s are not retained in TG latently infected with the S1L virus that lacks this epitope (Fig 8), agreeing with previously published data demonstrating that antigen is necessary to effectively maintain exogenously added memory CD8^+^ T cells in nonlymphoid tissues[18,53]. Thus, differential expression of viral proteins or peptides during contraction/latency appears to be one among a complex set of mechanisms that define the CD8^+^ TCR repertoire in latently infected TG. This indicates a requirement for ongoing antigen detection to maintain memory CD8^+^ T cells in TG during latency, and could imply ongoing recognition by non-gB-CD8s in the TG of S1L latently infected mice during latency. Various levels of antigen recognition during the contraction phase may also explain the faster CD8^+^ T cell contraction observed in the S1L infected TG at 16 dpi (Fig 7A). Although the subset of possible ganglionic memory CD8^+^ T cells is shaped early in the lymphoid tissue, it is likely that competition for antigen recognition is a major factor responsible for determining which CD8^+^ T cell populations are retained efficiently and which subsequently arise to prominence in latently infected TG.

We present several lines of evidence that suggest the subdominant CD8^+^ T cells in the TG are more functional than those in the WT latently infected TGs. Specifically, in stimulations with gB-null HSV-1 infected cells, the fraction of responding CD8s in TG latently infected with S1L was four-fold higher than the fraction of responding cells in TG infected with WT virus (Fig 7B). This was considerably more than that expected from a simple doubling of numbers due to the compensated response for loss of the gB-CD8s. Secondly, the totaling of all the CD8^+^ T cells responding to peptide stimulation in S1L latently infected TG was approximately twice that of the fraction of subdominant CD8s responding in a WT latent infection (Fig 10B and 10C). Indeed, the response seemed to account for most of the CD8^+^ T cells in those S1L latently infected TG. Finally, measuring of two major subdominant populations directed to RR1 by parallel tetramer staining and analysis in response to peptide stimulation indicate that proportionally more of these RR1-specific cells show multifunctionality than do those same T cells from a WT latent infection (Fig 10D). Taken together, these data suggest that eliminating the strongly immunodominant gB_498-505_ epitope increases the function of remaining CD8^+^ T cells specific for some non-gB_498-505_ HSV-1 epitopes.

We previously demonstrated that i) subdominant CD8^+^ T cells have a higher frequency and express higher levels of PD-1 and show less apoptosis when PD-1 ligation is blocked within latently infected TG [31]; and ii) in vivo IL-10R blockade significantly increased the number, but not the frequency of subdominant CD8^+^ T cells in TG latently infected with WT HSV-1, and increased their ability to block HSV-1 reactivation from latency in *ex vivo* TG cultures[30]. These characteristics are consistent with an exhausted phenotype. Here and in previous studies [30,31] we show that TG that are latently infected with WT virus contain functionally exhausted subdominant CD8s, but near fully functional immunodominant gB-CD8s. Thus, in TG latently infected with WT HSV-1 the frequency of cytokine producing cells following peptide stimulation was similar to the frequency of the corresponding tetramer positive cells in the immunodominant population, but significantly lower in subdominant populations. Using the same analysis we now show significantly reduced functional exhaustion of the subdominant CD8^+^ T cells in TG that lack the gB_498-505_ immunodominance. We further demonstrate a significantly increased number of multifunctional subdominant CD8^+^ T cells in TG latently infected with HSV-1 lacking the immunodominant epitope when compared to those infected with WT HSV-1.

It is unclear why i) subdominant CD8^+^ T cells become partially exhausted in latently infected TG while immunodominant gB_498-505_-specific CD8^+^ T cells retain nearly full functionality; and ii) subdominant CD8^+^ T cells show less functional exhaustion in latently infected TG that lack the immunodominant population. Given the recent evidence demonstrating the unregulated nature of antigen expression in periods of “animation” during latency[15, 17], effective CD8^+^ T cell surveillance of a variety of viral targets may provide better monitoring of neurons in the early stages of reactivation. There is likely an interplay between the amount of leaky viral antigen presentation during latency and the number of specific CD8^+^ T cells that can possibly “see” that antigen. However, this interplay may also explain the phenomena observed here- wherein there exist an apparently very low frequency of latently infected neurons that express viral proteins[16]. The absence of the gB_498-505_ recognition and subsequent responses by immunodominant T cells may itself influence sporadic antigen expression during latency in the TG, which may change how other T cells see such expression in general. This may in turn affect the function of the subdominant T cells. Furthermore, in TG infected with WT HSV-1, the frequency of gB_498-505_-specific CD8^+^ T cells is high, reducing the likelihood of any one cell receiving the multiple exposures to cognate antigen required for functional exhaustion over time. In contrast, the frequency of subdominant CD8^+^ T cell populations is low, increasing the likelihood of such repeat encounters with cognate antigen. In TG harboring latent S1L virus, the subdominant populations are greatly expanded, which should alter the dynamics and reduce the likelihood of repeat antigenic exposure. As such, the mechanisms underlying the functional differences may be very complex and multifactorial.

In summary, we have precisely defined the nature of a compensatory subdominant T cell response infiltrating the TG, a site of HSV-1 latency in our model, and show that it is due to expansion of the non-gB-CD8 populations. However, during latency, this population alters to a state where there are multiple CD8^+^ T cell populations that rise to co-dominance that seems to be in part due to ongoing antigen expression within the TG during contraction. Furthermore, these CD8s at latency demonstrate an increased functionality compared to their WT-induced counterparts. This results in a broader repertoire of functional HSV-specific CD8^+^ T cells, which should increase the set of antigenic targets that can be used by CD8^+^ T cells to prevent HSV-1 reactivation from latency.

## Materials and methods

### Virus and cells

Vero cells (ATCC, Manassas, Virginia), SV40-transformed B6 embryo fibroblast cell line B6WT3 (MHC-I compatible with C57BL/6 mice; [54]), and gB-Vero (Vero cells stably transfected with a plasmid expressing gB from the native gB promoter; obtained from Dr William Goins, University of Pittsburgh) were grown in Dulbecco's modified Eagle's medium (DMEM) supplemented with 10% fetal bovine serum (FBS), penicillin-G (100 units/ml), streptomycin (100 mg/ml) and fungizone (250 mg/ml). All HSV-1 are based on the KOS strain originally obtained from a master stock obtained from P Schaffer. This strain was recently sequenced (GenBank JQ780693.1) [55]

Pseudorabies virus (PRV) used in this study were generated using a Becker-strain bacteria artificial chromosome (BAC) (kind gift from Lynn Enquist, Princeton University) [56] PRV expressing HSV-1 gB_494-509_ in a thymidine kinase (TK) knockout virus. This was developed in the E. Coli strain GS1783 by placing a kanamycin-resistance cassette containing monomeric red fluorescent protein in frame with TK at site 59,595 (Genbank JF797219.1) followed by a CMV promoter driving expression of 4 repeats of HSV-1 gB_494-509_. This cassette was amplified using the following primers: Forward, 5'-GCGGCAACCTGGTGGTGGCCTCGCT GGACCCGGACGAGCACATG GCCTCCTCCGAGGACGTCAT-3'; Reverse, 5’- TCAGGTAGCGCGACGTGTTGACC AGCATGGCGTAGACGTTCCTCG CGAGGGATCGGCTAGAGTC -3’ and inserted into the PRV BAC using recombineering [57]. The kanamycin-resistance cassette was resolved using two-step red-mediated recombination, with a resultant disruption of the TK gene. PRV was propagated to high titer in PK15 (porcine kidney 15, ATCC CCL-33) epithelial cells, purified as done for HSV, and gB-peptide expression confirmed by stimulation of gB-specific T cells, as described below.

### Derivation of HSV-1 with gB_498-505_ mutations

DNAs were amplified by polymerase chain reactions (PCR) using a proofreading polymerase [Expand (Roche) or Primestar (Takara)] with “Hot start” conditions and in reactions containing 4% DMSO, as detailed previously [47]. A plasmid was derived in which genomic recombination into HSV-1 would replace the approximately N terminal half of the gB coding sequence with eGFP, using a pUC19-based plasmid (gC Rescue construct, Fig 1) detailed previously [47] containing HSV-1 DNA from 54,475bp to 56,464bp (sequences given in reference to HSV-1 KOS Genbank JQ780693.1) containing the gB promoter and partial gB coding region. The construct had a unique EcoRI flanking at 54,475 and unique HindIII site flanked 56,464, and a unique AvrII site at position 58,476bp, immediately upstream of the gB ORF start and designed not to alter the coding of the upstream UL28 protein. The AvrII-EcoRI fragment of this plasmid was replaced with a PCR fragment encoding gB residues 507 to the stop codon, generated using primers 5’-GCGCCTAGGCTCGGATCCCAGTTTACGTACAAC-3’ and 5’-GAGCGGAATTCATTTACAACAAACCCCCCATCA-3’ (restriction sites underlined). This construct (termed pgBp-gBend) was then further modified by inserting a PCR-amplified fragment containing eGFP using the primers: 5’ CCCTAGGCTACCTGACGGCGGGCACGACGG 3’ and 5’ CGTAGGATCCTTACTTGTACAGCTCGTC 3’. The BamHI-AvrII fragment was inserted into BamHI-AvrII digested pgBp-gBend, resulting in the flanking of eGFP by the gB promoter sequences, and sequence encoding gB residues 507–904 (Fig 1). When this linearized plasmid was then cotransfected with infectious HSV-1 DNA on gB-Vero cells, recombinant viruses showing eGFP positivity were selected and grown on complementing gB-Vero cells. Infectious HSV-1 KOS DNA was obtained using methods outlined previously [47]. Recombinant viruses (HSV-1 gB-null-EGFP) were then verified for correct DNA insertion by DNA sequencing of the junctions and by Southern blot analyses.

Plasmid gBp-gBend was digested with AvrII-SnaBI to remove the EGFP cassette, and replaced with PCR amplified sequence encoding gB residues 1–509 with altered residues in the gB SSIEFARL motif (Table 1). The primers shown in Table 1 were used in conjunction with the primer 5’ GCCCTAGGCTACCTGACGGGGGGCACGACGGGCCCCCGTAG 3’. Each PCR was then cloned as an AvrII-SnaBI fragment and sequence verified. Linearized plasmids were then individually cotransfected with HSV-1 gB-null-EGFP infectious DNA on Vero cells, and HSV-1 recombinants replicating on Vero cells, lacking GFP expression, and containing the mutations in the gB_498-505_ region were plaque-purified. We also derived expression plasmids for each epitope-mutated gB protein by placing the AvrII-EcoRI fragment containing the entire altered gB protein coding region into the vector pEGFP-C3, digested with NheI and EcoRI to place gB under the control of the human cytomegalovirus (hCMV IE) Immediate early promoter and SV40 polyadenylation signals.

### Animal infection, tissue preparation, and *ex vivo* reactivation studies

Six to eight week old female C57BL/6 mice (Purchased from Jackson Labs) or gB-T1 mice [58] were used in these studies. HSV-1 infection by the ocular route was detailed previously [47], Briefly, corneas of anesthetized mice were scratched using a 30g needle with care not to enter the basement membrane. HSV-1 (1 x 10^5^ PFU of purified HSV-1 in 3 μl RPMI media) was then applied and massaged into the eye, animals were then allowed to recover from anesthesia and returned to housing. Infections of the mouse flank were at a small (5mm x 5mm) region of skin denuded of hair. 1 x 10^6^ PFU of purified HSV-1 was applied to abraded skin. Tissues for subsequent flow cytometry analyses were obtained from anesthetized mice injected i.p. with 0.3 ml of 1000 U/ml heparin (Sigma-Aldrich, St. Louis, MO) and then euthanized by exsanguination. Lymph nodes, TG and/or spleen were removed and subsequently digested in RPMI containing 10% fetal bovine serum and 400 U/ml collagenase type I (Sigma-Aldich) for 45–60 minutes at 37°C. Tissues were mechanically dissociated and triturated into single-cell suspensions and then filtered through a 40 μm nylon cell strainer (BD Biosciences, Bedford, MA). Spleens were treated with red blood cell lysis buffer (BD Pharm Lyse) for three minutes prior to analyses.

For *ex vivo* reactivation, TG samples were first rendered into single cell suspensions as just detailed, but without filtering. In cases where endogenous CD8^+^ T cells were depleted, we used antibody/complement mediated lysis using Low-Tox M rabbit complement (Cedarlane) and anti CD8, as previously described[20]. The efficiency of depletion was assessed by flow cytometry and was considered effective if >95% of the CD8^+^ T cells were depleted. Mock depleted suspensions used an IgG isotype control under the same conditions. Single-cell TG suspensions were then plated at one-fifth TG equivalents per well in 48-well culture plates in 400 μl of DMEM containing 10% FBS, 10 mM HEPES buffer, 10 U/ml recombinant murine IL-2 (R&D Systems), and 50 μM 2-mercaptoethanol. Where indicated, cultures were supplemented with exogenous expanded gB-CD8s made as described previously at 2 x 10^4^ CD8^+^ T cells/well [24]. TG cultures were monitored for reactivation by testing 50 ul culture supernatant fluid for live virus by standard viral plaque assays as previously described [20]. The 50ul was replaced each time with fresh media. Supernatants were tested every two days for a total of ten days in culture. Data are represented as the percent of wells that were positive for viral reactivation.

### CD8^+^ T cell expansion, stimulation and flow cytometry

CD8^+^ T cells specific for gB_498-505_ were expanded from TG preparations taken 8 days post infection (dpi) from mice infected with HSV-1, as detailed previously [24], or with collagen-dissociated suspensions of TGs. Cultures were maintained for up to 10 days, followed by MACS bead purification of the CD8^+^ T cells. Resulting populations were >95% CD3^+^, CD8^+^, and positive for gB_498-505_/H-2K^B^ tetramer. For preparing target B6WT3 fibroblasts, the (WT) and mutant gB proteins were expressed in B6WT3 (1 x 10^5^ cells) transfections with 5 μg of plasmids expressing each gB protein or the epitope mutant protein under the CMV-IE promoter. At 24 hours post-transfection, co-cultures for stimulations were established, with 5 x 10^4^ expanded gB-CD8^+^ or CD8^+^ T cells obtained from the TG of HSV-1 infected gB-T1 mice added per 1 x 10^5^ target B6WT3 cells. Co-cultures were maintained for 6 h in the presence of Brefeldin A, and then subsequently stained for surface CD45, CD8 and intracellular IFNγ and/or TNFα as markers of activation.

T cell phenotypic characterization by flow cytometry was performed essentially as detailed previously [26]. Single cell suspensions of TGs and spleens were stained with antibodies to CD45, CD3, and CD8α, and with tetramers for 1 hr at room temperature prior to fixing for 20 minutes with Cytofix/Cytoperm (BD Biosciences, Bedford, MA). Washed cells were then analyzed by flow cytometry. CD8^+^ T cell recognition of HSV-1 target antigens was determined by pulsing cultured B6WT3 fibroblasts with the respective peptide [26] at a concentration of 1 μg/ml for 30 min at 37°C/5% CO_2_. Alternatively, B6WT3 fibroblasts were infected for 6–12 hours at an MOI of 5 with recombinant HSV-1 or PRV, and then virus was UV-inactivated prior to stimulation. The dispersed TG or spleen cells were added to peptide-pulsed or infected fibroblasts in the presence of Brefeldin A and anti-CD107a (BD clone 1D4B) for 6 hr at 37°C/5% CO_2_. After stimulation, cells were stained for surface expression of anti-CD45 and CD8α, permeabilized and fixed using Cytofix/Cytoperm and then subjected to intracellular stain for IFNγ and TNFα. The peptides used in this work were detailed previously [26]. For each peptide, both TGs from a minimal total of five mice per peptide were separately analyzed for reactivity.

### Antibody reagents and flow cytometry

Phycoerythrin (PE)-conjugated or BV421-conjugated H-2K^b^ tetramers complexed with the gB_498-505_, RR1_982-989_, or RR1_822-829_ peptide were provided by the National Institute of Allergy and Infectious Diseases Tetramer Core Facility (Emory University Vaccine Center, Atlanta, GA). Efluor-450 conjugated anti-CD3 (clone 17A2) was purchased from eBioscience. Pacific-Blue-conjugated anti-CD8α (clone 53–6.7), APC-conjugated anti- IFNγ (clone XMG1.2), PerCP-conjugated anti-CD45 (clone 30-F11), PE-Cy7-conjugated anti-TNFα (clone MP6-XT22), APC-conjugated anti-granzyme B (clone GB11), and BD Cytofix/Cytoperm Fixation/Permeabilization Solution Kit were purchased from BD Pharmingen (San Diego, CA). The appropriate isotype control antibodies were purchased from the same company used for the reactive antibody and used as controls for intracellular staining. All flow cytometry samples were collected on BD FACSAria cytometer and analyzed by FACSDiva and/or FlowJo software.

### Quantitative real-time PCR

HSV-1 genome copy number in infected TG was determined by quantitative real-time PCR as previously described using primers that recognize the sequences of the gH gene [59].

### Statistical analysis

All statistical analyses were performed using GraphPad Prism software package. The specific statistical applications are shown in the legends to each figure.

### Ethics statement

All animal experiments were conducted in accordance with protocol # 15076444, approved by the University of Pittsburgh Institutional Animal Care and Use Committee. This protocol meets the standards for humane animal care and use as set by the Animal Welfare Act and the NIH Guide for the Care and Use of Laboratory Animals.
